# Carcass appearance does not influence scavenger avoidance of carnivore carrion

**DOI:** 10.1038/s41598-022-22297-8

**Published:** 2022-11-07

**Authors:** Miranda J. Butler-Valverde, Travis L. DeVault, Olin E. Rhodes, James C. Beasley

**Affiliations:** 1 Savannah River Ecology Laboratory, Warnell School of Forestry and Natural Resources, University of Georgia, P.O. Box Drawer E, Aiken, SC 29802 USA; 2 Savannah River Ecology Lab, University of Georgia, P.O. Box Drawer E, Aiken, SC 29802 USA

**Keywords:** Ecology, Behavioural ecology, Community ecology

## Abstract

The selection or avoidance of certain carrion resources by vertebrate scavengers can alter the flow of nutrients in ecosystems. Evidence suggests higher trophic level carrion is scavenged by fewer vertebrate species and persists longer when compared to lower trophic level carrion, although it is unclear how scavengers distinguish between carcasses of varying species. To investigate carnivore carrion avoidance and explore sensory recognition mechanisms in scavenging species, we investigated scavenger use of intact and altered (i.e., skin, head, and feet removed) coyote—*Canis latrans* (carnivore) and wild pig—*Sus scrofa* (omnivore) carcasses experimentally placed at the Savannah River Site, SC, USA. We predicted carnivore carcasses would persist longer due to conspecific and intraguild scavenger avoidance. Further, we hypothesized visually modifying carcasses would not reduce avoidance of carnivore carrion, given scavengers likely depend largely on chemical cues when assessing carrion resources. As expected, mammalian carnivores largely avoided scavenging on coyote carcasses, resulting in carnivore carcasses having longer depletion times than wild pig carcasses at intact and altered trials. Therefore, nutrients derived from carnivore carcasses are not as readily incorporated into higher trophic levels and scavengers largely depend on olfactory cues when assessing benefits and risks associated with varying carrion resources.

## Introduction

Carrion is an important source of nutrients for many species including vertebrates, invertebrates, microbes, and plants^1–3^. Species across all biological kingdoms benefit from carrion, and as a result carrion derived nutrients are directly reincorporated into ecosystems across all trophic levels^4^. For example, vertebrate scavenging leads to the retention of nutrients at upper trophic levels that are subsequently redistributed more widely on the landscape, whereas carcass nutrients sequestered by lower trophic level decomposers and invertebrates are usually redistributed in the carcass vicinity^5–7^. Vertebrate scavenging communities are diverse, and a myriad of environmental, behavioral, and carcass attributes influence carcass use by various taxa^8–10^, including carcass avoidance and preference^11,12^. Due to the complex feeding links and widespread availability of carrion, vertebrate scavenging is an important contributor to ecosystem structure and function^2,3,13^. To better understand food web links and nutrient cycling, it is imperative to determine factors that influence vertebrate scavenging dynamics, including carrion avoidance behaviors. Although some studies have investigated potential factors contributing to avoidance behaviors, such as risk of contracting a disease, these assumptions and the mechanisms through which scavengers distinguish carcass types have not been thoroughly assessed.

One factor that can contribute to carrion avoidance is carcass type, as the species or taxa of a carcass has been shown to affect the rate of carrion consumption and scavenger community composition^12,14–16^. Studies that have used both carnivore and herbivore carrion have found carnivore carcasses are generally scavenged less frequently and by fewer vertebrate species^12,17,18^. Reduced scavenging rates of higher trophic level carrion might reflect an effort to lessen exposure to diseases by avoiding scavenging on conspecifics or species within guilds, as the risk of pathogen transmission increases when consuming phylogenetically similar species^12,19^. However, cannibalism and consumption of related taxa are reported broadly in the literature^20–22^, suggesting intrinsic (e.g., evolutionary adaptations) or extrinsic (e.g., limited food availability) factors may increase species’ affinity to scavenge on conspecifics or more closely related species^14,23^.

Although emerging evidence suggests carcass type is linked to carrion avoidance and preference patterns, the mechanisms through which scavengers distinguish carcasses of different species have not been thoroughly explored. Species have evolved diverse sensory organs and capabilities (e.g., vision, audition, and chemoreception) to identify food, potential risks (e.g., predators), and conspecifics including kin^24,25^. The extent to which species rely on chemical and visual cues in assessing palatability and risk associated with carrion is likely similar to their assessment of other food sources (excluding obligate scavengers), given interspecific differences in sensory capabilities and foraging strategies. It is also probable that species use multiple senses to varying degrees to assess palatability of carrion, influenced by a hierarchy of cues (e.g., visual, odor, touch). For instance, volatile compounds produced by microbial decomposers act as olfactory signals that lead vertebrate scavengers to carrion, but also indicate carcass palatability, depending on the concentration of repellent toxins present^1,26^. However, the extent to which species rely on visual and chemical cues in their decision process to scavenge among carcass types is largely unknown.

Several scavenging studies have used carcasses that were not fully intact including partially or fully skinned, eviscerated, and/or head and feet removed^18,27,28^. Yet, few of these assessed how these alterations, and thus external characteristics and associated chemical stimuli, influence scavengers’ feeding decisions (but see Moleón et al.^12^ and Selva et al.^29^), and most had limited sample sizes. Moleón et al.^12^ experimentally tested whether scavengers would discriminate between similarly sized barbary sheep—*Ammotragus lervia* (herbivore) and red fox—*Vulpes vulpes* (carnivore) carcass portions that had all external characteristics removed. Their findings revealed scavengers avoided carnivore carrion despite the lack of distinguishing morphological features or fur, suggesting olfactory cues play a large role in scavengers’ differentiation between carcass types. Conversely, Carr^30^ assessed cannibalism in laboratory Norway rats (*Rattus norvegicus*) and found rats did not discriminate between scavenging on a house mouse (*Mus musculus*) or a conspecific when carcasses were skinned, whereas when offered intact carcasses cannibalism was not observed. Although these laboratory results should be considered with caution given they are the product of laboratory-controlled experiments which may not reflect behaviors expressed in natural populations, they do suggest visual cues or chemical stimuli originating from the skin and fur of a carcass may affect scavenger consumption decisions.

By experimentally placing intact and physically altered (i.e., carcasses were skinned completely and their skull and feet were removed) coyote—*Canis latrans* (carnivore) and wild pig—*Sus scrofa* (omnivore) carcasses on the landscape, we aimed to investigate carnivore carrion avoidance behaviors and explore the extent to which scavengers rely on external carcass characteristics to distinguish between carcass types. In addition, we conducted trials with coyote and pig carcasses placed at the same location to consider carnivore carrion as an influential aspect in the ‘landscape of fear’, due to perceived predation risk, and ‘landscape of disgust’, due to infection risk^31,32^. We tested the following hypotheses: (1) conspecifics strongly avoid cannibalizing, (2) carnivore carcasses persist longer and are generally avoided by intraguild scavengers (i.e., carnivorous mammals), and (3) avian scavenger diversity is higher at carnivore carcasses due to the longer persistence of carnivore carrion and the similar propensity for avian scavengers to consume carnivore and omnivore carcasses given the lower risk of contracting diseases from mammalian carcasses. In addition, we anticipated the removal of external characteristics (i.e., skinning and removing the head and feet) would minimally influence scavenging dynamics, given olfactory cues would largely still be present. Therefore, we predicted our hypotheses would apply for both intact and altered carcass experiments.

## Results

We deployed 136 carcasses (i.e., 68 coyotes and 68 wild pigs; 40 intact and 28 altered carcasses for each species) from February to June 2021. Three cameras malfunctioned mid-trial at intact trials (one wild pig paired with a coyote, and two coyote trials) and one failed altogether (one coyote paired with a wild pig trial). For trials that failed prematurely, we used all available data collected before the failure for analyses, which resulted in different sample sizes among some of our analyses. The trial that failed altogether was excluded from the dataset.

**Figure 1:**
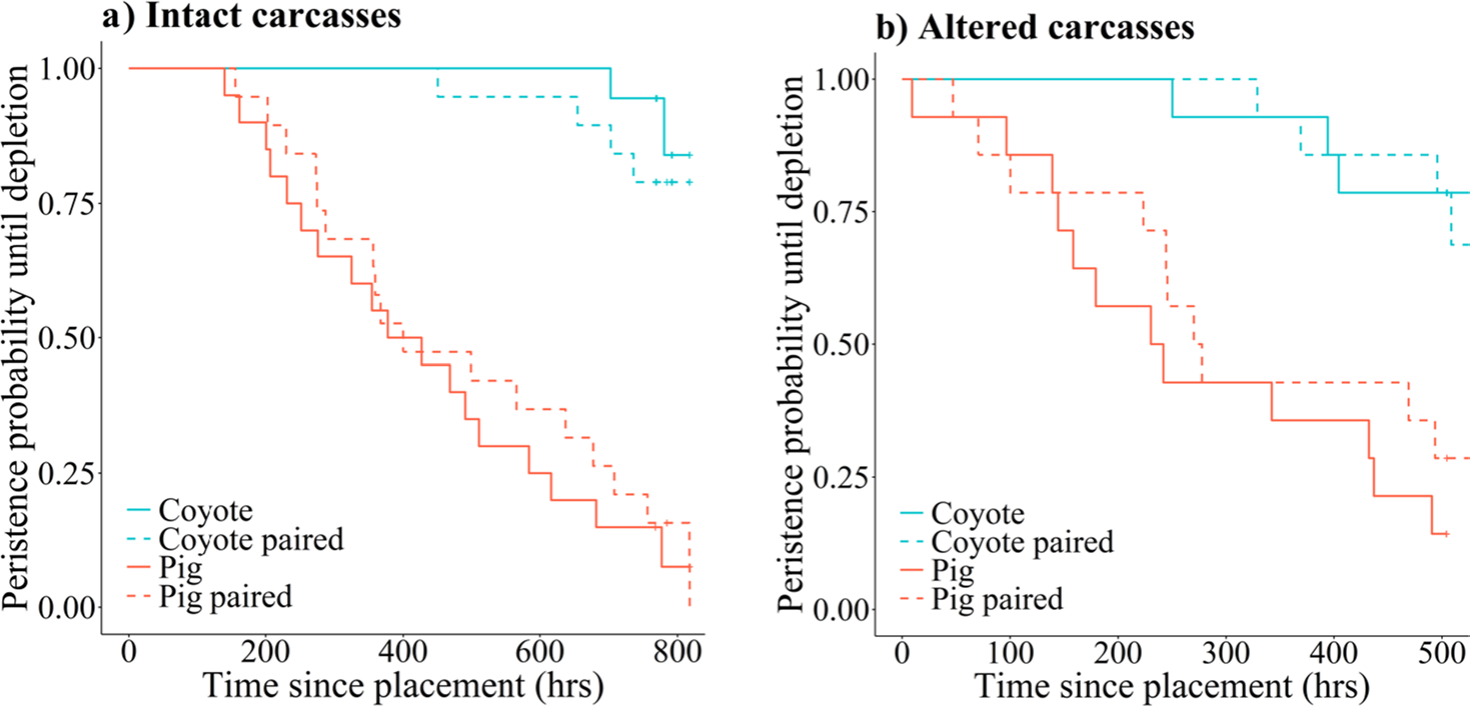
Kaplan-Meir survival function estimate of the probability of the four carcass configurations persisting on the landscape until depletion for (**a**) intact carcass trials and (**b**) altered carcass trials conducted February–June 2021 at the Savannah River Site, Aiken, SC, USA. Censoring is indicated by the plus sign tick marks.

Mean time to first investigation was similar across carcass configurations for both intact and altered trials, suggesting there was no difference in detectability among carcasses (intact: chi-square = 1.54, df = 3, *P* = 0.67; altered: chi-square = 0.54, df = 3, *P* = 0.91; Fig. S1). The Mantel-cox test revealed there was also no difference in time to first scavenging among carcass configurations for intact (chi-square = 6.52, df = 3, *P* = 0.09) or altered (chi-square = 0.34, df = 3, *P* = 0.95) carcasses (Fig. S1). In contrast, the Mantel-cox test revealed persistence times varied among carcass configurations for both intact (chi-square = 54.97, df = 3, *P* < 0.001) and altered (chi-square = 25.49, df = 3, *P* < 0.001) carcasses (Fig. 1). The Cox-proportional hazards model further revealed that mass affected carcass persistence for intact carcasses, with larger carcasses persisting longer on the landscape (Hazard Ratio [HR] 0.90, 95% confidence interval [CI] 0.83–0.98, *P* = 0.01), but did not influence persistence for altered carcasses (HR 0.98, 95% CI 0.81–1.18, *P* = 0.80). Carcass persistence was different between the reference carcass type (coyote) and wild pig (intact: HR 27.20, 95% CI 6.20–119.37, *P* < 0.001; altered: HR 10.10, 95% CI 2.43–41.92, *P* = 0.001) and paired wild pigs (intact: HR 19.25, 95% CI 4.42–83.78, *P* < 0.001; altered: HR 6.83, 95% CI 1.84–25.40, *P* = 0.004), whereas paired coyote trials (intact: HR 1.88, 95% CI 0.34–10.25, *P* = 0.47; altered: HR 1.65, 95% CI 0.39–7.01, *P* = 0.50) were not different than coyote. Mean first investigation, first scavenging, and carcass persistence times are summarized for the carcass configurations in Table 1.

**Table 1:** The minimum, maximum, and mean persistence times (hours), and associated standard deviations, calculated across carcass configurations for intact and altered carcasses based on scavenging trials completed between February–June 2021 at the Savannah River Site in Aiken, SC, USA.

Event	Carcass configuration	Carcass condition							
		Intact				Altered			
		Min	Max	Mean ± SD	*n*	Min	Max	Mean ± SD	*n*
Investigate	Coyote	8.4	159.1	70.3 ± 50.7	20	0.5	61.7	20.9 ± 22.9	14
	Coyote paired	5.6	253.9	86.8 ± 74.4	19	0.3	44.0	17.6 ± 16.7	14
	Wild pig	4.3	352.5	92.3 ± 105.7	20	0.4	72.3	18.3 ± 25.6	14
	Wild pig paired	5.6	253.9	85.5 ± 72.7	20	0.3	44.0	17.6 ± 16.7	14
Scavenge	Coyote	27.7	526.3	198.3 ± 158.2	20	0.5	122.6	34.2 ± 40.0	14
	Coyote paired	7.5	389.3	194.4 ± 129.9	19	0.3	94.3	32.0 ± 32.3	14
	Wild pig	12.7	530.0	249.3 ± 152.4	20	0.4	504.1	61.9 ± 131.3	14
	Wild pig paired	6.8	361.5	160.9 ± 104.2	20	0.3	97.3	33.6 ± 33.9	14
Persistence	Coyote	702.6	817.7	781.9 ± 27.8	18	250.3	549.7	497.2 ± 88.2	14
	Coyote paired	450.3	817.7	761.8 ± 86.4	19	328.9	549.3	502.5 ± 68.7	14
	Wild pig	140.3	817.4	433.2 ± 216.5	20	9.1	504.3	279.3 ± 168.8	14
	Wild pig paired	156.0	817.6	482.6 ± 228.0	19	47.0	548.2	324.5 ± 182.4	14

The Virginia opossum (*Didelphis virginiana*) and turkey vulture (*Cathartes aura*) were the most frequent scavengers at the greatest number of intact (32 and 26, respectively) and altered (17 and 34, respectively) trials (Tables S1 and S2). Further, when comparing the number of scavenging events among unpaired and paired carcass configurations separately, Virginia opossums and turkey vultures scavenged coyote carcasses more times than wild pig carcasses for both intact and altered trials (Tables S3 and S4). Despite frequent scavenging by Virginia opossums and turkey vultures, coyotes were the last scavenger to feed on the most intact and altered carcasses (24 and 14, respectively). In addition to these summary aspects, coyotes cannibalized at six altered carcasses (nine scavenging events) in comparison to one intact carcass (one scavenging event). For altered coyote carcasses, these scavenging events ranged from 10 to 17 days since trial initiation, with a median of 11 days, and in the case of the intact coyote, cannibalism occurred 28 days after the start of the trial.

**Figure 2:**
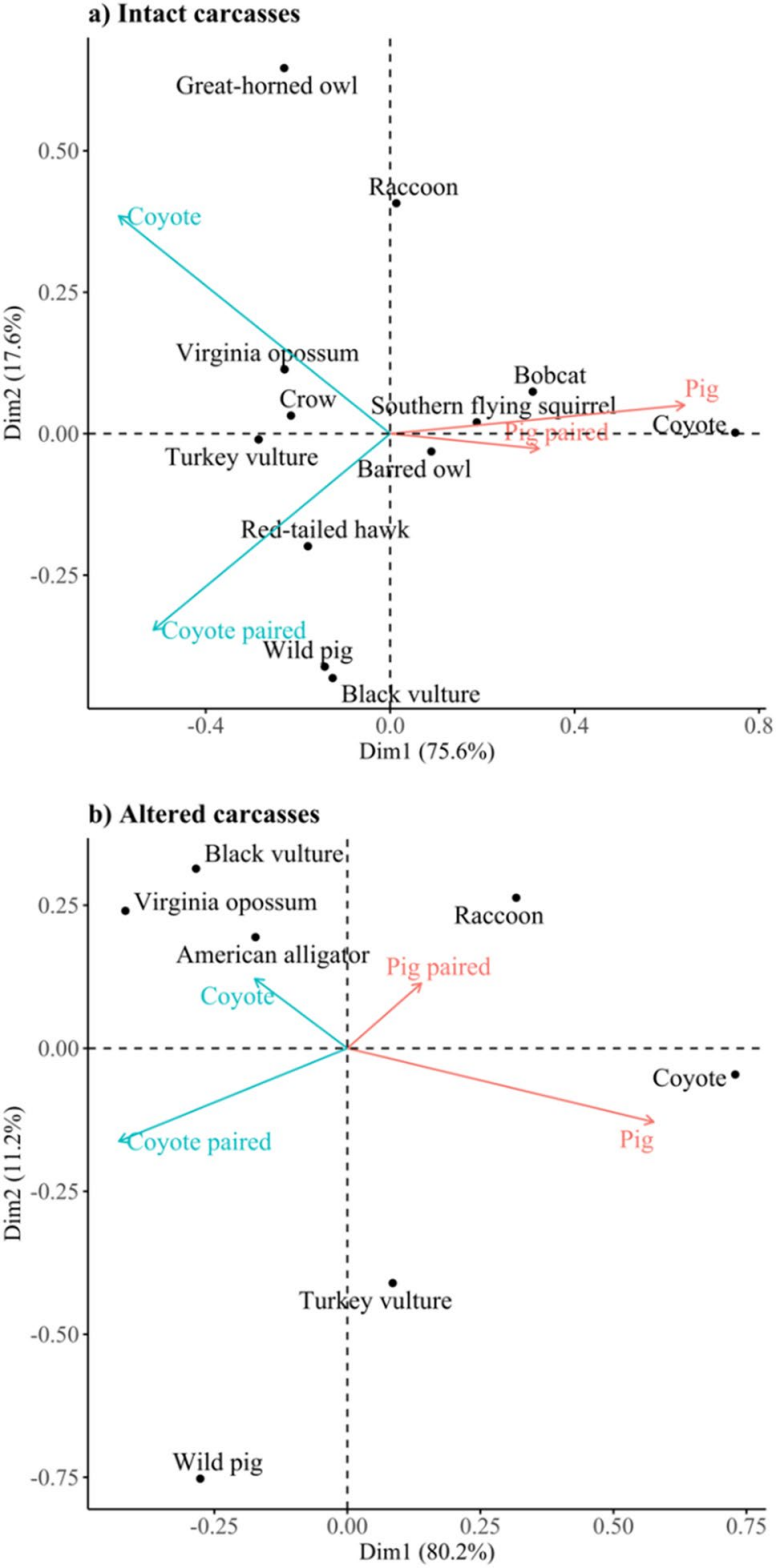
Contribution biplot depicting the result of the correspondence analysis for (**a**) intact carcasses and (**b**) altered carcasses investigating vertebrate scavenger species (black dots) occurrence on the four carcass configurations (red arrows) with variance shown in paratheses based on scavenging trials completed in February–June 2021 at the Savannah River Site in Aiken, SC, USA.

The correspondence analysis revealed scavenger assemblages of unpaired wild pig and coyote carcasses were largely distinct from each other for both intact and altered trials given the degree of separation in the biplots (Fig. 2). For intact trials, wild pig and paired wild pig carcasses had more similar scavenging communities than other carcass configurations. Intact coyote carcasses had different assemblages between coyote only and coyote carcasses paired with wild pigs, as raccoons (*Procyon lotor*) and great-horned owls (*Bubo virginianus*) only scavenged unpaired coyote carcasses (Table S1). The correspondence analysis also revealed a greater diversity of avian scavengers associated with intact coyote carcass assemblages than intact wild pigs. Altered carcass configurations had distinct scavenger assemblages (Fig. 2b), with carnivore carrion being scavenged dissimilarly than omnivore carrion. Virginia opossum and wild pig were more associated with the scavenger assemblages of coyote carcass configurations, whereas the raccoon and coyote were more associated with wild pig carcass configurations.

At intact trials, 12 vertebrate species were documented scavenging intact carcasses, whereas 7 species were documented scavenging altered carcasses (Tables S1 and S2). These numbers do not include non-sciurid rodent species, given smaller vertebrate species were not reliably detected at all carcasses. Excluding rodent scavenger diversity may have affected our results given many rodents are scavengers^10,14^, therefore future studies should incorporate more sensitive camera technology to more thoroughly capture the vertebrate scavenging community. Scavenging species richness varied from 1 to 5 species at intact carcasses and 0 to 4 species at altered carcasses. For intact trials, carcass mass did not influence scavenger richness (*P* = 0.62; Table S5); however, scavenger species richness was greater for paired wild pig carcasses ($\overline{x}=$ 3.21), the reference group, than for unpaired wild pig carcasses ($\overline{x}=$ 2.15; *P* = 0.04). There were no other significant interactions between carcass configurations and scavenger richness for intact trials (coyote—$\overline{x}=$ 2.22; *P* = 0.07, paired coyote—$\overline{x}=$ 2.42; *P* = 0.15). Scavenger richness did not differ among altered carcass configurations (coyote, $\overline{x}=$ 3.14; paired wild pig, $\overline{x}=$ 2.93; paired coyote, $\overline{x}=$ 2.64; and wild pig, $\overline{x}=$ 2.00), and carcass mass did not influence scavenger species richness (Table S5).

When considering joined species richness at paired carcasses, there was a difference in scavenger species richness among carcass configurations for both intact (chi-squared = 17.168, df = 2, *P* < 0.001) and altered (chi-squared = 9.647, df = 2, *P* = 0 0.008) trials. The Dunn’s multiple comparison test revealed there was no difference in species richness between intact, unpaired coyote and wild pig carcasses (z = 0.30, *P* = 1.000); however, intact, paired trials had greater combined scavenger richness than both unpaired coyote (z = − 3.40, *P* = 0.002) and wild pig carcasses (z = 3.78, *P* < 0.001). For altered trials, scavenger richness was greater for paired trials than for unpaired wild pig carcasses (z = 2.95, *P* = 0.009), but there were no other differences in scavenger richness among carcass configurations (coyote—paired: z = − 0.64, *P* = 1.00; coyote—wild pig: z = 2.3, *P* = 0.063).

The model assessing the influence of carcass condition and temperature on pig carcass persistence met the assumptions of a generalized linear model (GLM)^33^. At pig carcass trials, temperatures (*P* = 0.52) and carcass condition (*P* = 0.08) were not found to influence carcass persistence (Table S6). In contrast, at coyote trials, higher mean air temperatures were associated with shorter persistence times (*P* = 0.02) and intact carcasses were found to persist longer than altered ones (*P* = 0.001; Table S6), although the model for the coyote data did not meet the assumptions of the GLM despite exhaustive transformations and attempts to fit a better distribution and link function^33^. Thus, to validate the finding of different persistence times between altered and intact carcasses, we additionally performed a one-sided Wilcoxon rank sum test on the coyote data using the R package *coin*^34^. These results further support the finding that intact carcasses persisted longer than altered ones (z = 6.56, *P* < 0.0001). In addition, paired and unpaired altered coyote carcasses collectively were cannibalized more than intact paired and unpaired coyote carcasses (*P* = 0.037, Fisher’s exact test).

## Discussion

In support of our second hypothesis, we found coyote carcasses persisted longer and were less likely to be entirely scavenged by vertebrates than wild pig carcasses, a pattern that was consistent between intact and altered carcass trials. These results provide further evidence that use of carcasses by facultative scavengers is complex and influenced by many factors, most likely including disease risks, nutritional benefits, resource availability, and individual and species preferences^12,14,23^. In particular, differential periods of carcass persistence between carrion of different trophic levels may have ramifications for necrophagous insect communities, as carcasses that are rapidly consumed by vertebrates may serve as ecological sinks for these species^17,35^. Indeed, previous studies have indicated larval necrophagous insects may predominately obtain nutrients from higher trophic level carrion, likely due to a reduction in vertebrate scavenger competition and thus longer persistence time of carnivore carcasses^17,35^. However, larger carcasses also have been shown to support multi-guild invertebrate communities, given their longer persistence times, thus large non-carnivore carrion likely also represents an important source of nutrients to insects^36^. Future carrion studies should incorporate comparisons of both invertebrate and vertebrate scavenger richness and abundance across carcasses of varying trophic levels^17^. In addition, the impacts of changes in carnivore distribution and abundance on other potential beneficiaries (i.e., microbial and plant) of longer-persisting carnivore carcasses, and the resulting impacts to scavenging community dynamics and ecosystem services provided by carrion removal should also be investigated (but see Cunningham et al.^37^).

Scavenger species richness did not greatly differ between higher and lower trophic level carcass types for intact or altered trials. However, at intact trials scavenger richness was significantly less at wild pig carcasses when compared to wild pig carcasses paired with coyotes. This suggests the presence of an intact coyote carcass did not deter scavengers from feeding on the intact wild pig carcasses at paired trials, and that the added carrion biomass of a second carcass may attract more scavengers to an area, increasing scavenger species richness at wild pig carcasses. Further, coyote carcasses may present distinct synergistic cues that attract scavengers compared to wild pigs, potentially contributing to greater scavenger richness at multi-species carcass sites. In altered trials, the combined scavenger species richness at paired carcasses also was greater than wild pig carcasses alone and at intact trials species richness of paired carcasses was greater than at both single carcass types, suggesting a higher diversity of carcass types in one location may support more diverse scavenger communities.

Some species, such as Virginia opossum and turkey vulture, took advantage of the longer persisting carnivore carrion and readily scavenged coyote carcasses, which supports other findings that indicate certain species are less likely to discriminate among carcass types^16^. Further, observational findings support our third hypothesis given some bird species scavenged on coyote carcasses at more trials (i.e., crow—*Corvus* spp. and great-horned owl; Table S1) or more frequently (i.e., turkey vulture; Table S2) than on wild pig carcasses. This is likely due to reduced vertebrate scavenger competition at coyote carcasses and not the longer persistence of carnivore carrion like we predicted, given bird species predominantly scavenged on coyotes during the first half of trials. Therefore, mammal carnivore carrion may represent an important food source for some vertebrate scavengers in our study system. Differences in vertebrate scavenger use of carcasses across trophic levels might have been more distinct had we used herbivore instead of omnivore carrion in our comparison with carnivore carrion.

Bobcats (*Lynx rufus*) entirely avoided scavenging on intact coyote carrion (paired and unpaired), whereas they consumed a large proportion of the intact wild pig carcasses they scavenged (Table S1), and coyotes generally avoided scavenging on conspecifics for intact carcasses. Together, these findings support our first hypothesis and the findings of other studies that carnivore carrion is typically avoided by conspecific and intraguild species^12,16,17,28^. More similar species (i.e., phylogenetically and ecologically) harbor comparable parasite assemblages^38^ and therefore it is assumed that mammalian carnivores likely avoid cannibalizing and scavenging on other carnivores due to perceived disease risks^28^. However, macroparasites such as *Trichinella* spp. (phylum Nematoda) and *Toxoplasma gondii* (phylum Apicomplexa) are widespread, can be transmitted through scavenging routes, and infect a variety of hosts, including coyotes and wild pigs^39–41^. Consequently, scavengers are at risk of being exposed to these parasites when consuming a variety of carrion types.

Some pathogens predominately affect carnivore species such as canine distemper virus and canine parvovirus, and although these viruses are largely thought to be transmitted antemortem, it has been suggested they might also be transferred through scavenging infected carcasses^42,43^. Therefore, carnivores may avoid scavenging within their guild due to potential health risks from more specialized illnesses like canine distemper and parvovirus. Further, carnivores might bioaccumulate more diseases due to their diverse diets^44,45^. In addition, species in the order Carnivora (307 extant species^46^) collectively carry more zoonotic diseases than the largest order of mammals, rodents (2,623 extant species^46^)^47^. The order Carnivora also has lost immune response pathways, potentially resulting in pathogens evading host detection^48^. Factors driving present day carnivore carrion avoidance behaviors may be multifaceted, but likely are driven by adaptions to reduce infection exposure^49,50^. Further, to the best of our knowledge, no carrion study has assessed the potential influence deleterious persistent organic pollutants and heavy metals bioaccumulating in higher trophic level species has on scavenging dynamics.

Although coyotes mostly avoided scavenging on conspecifics, there were mixed findings given only one incidence of cannibalism for intact coyote carcasses and nine (i.e., six trials) for altered carcasses. We note that cannibalism of altered carcasses predominantly consisted of coyotes scavenging on bones. This could be the result of several potentially compounding factors. For example, the increased occurrence of cannibalism documented at altered carcasses could be due to the modifications of these carcasses, which may have rendered them less distinguishable as coyote carcasses, especially given chemical cues likely subsided later in trials when most coyote scavenging events occurred. Further, removal of the heads of these carcasses left no skull present at the carcass site, which could be an additional visual cue scavengers use to distinguish carcasses. Alternatively, coyotes may have been willing to scavenge on altered carcasses given bones were accessible and mammal bones have a higher energy content than muscle tissue, largely due to high fat content^51^. Coyotes may be more willing to eat carnivore carcasses, including bones, at later stages of decomposition, given reduced parasite exposure risks^52^. In addition, freezing carcasses damages tissues and inhibits internal bacterial activity altering the decomposition process and arthropod colonization^53^, which likely also affects the olfactory cues omitted. Therefore, because altered carcasses were thawed and refrozen, whereas intact carcasses were not, this may have affected the palatability and scavenger use of altered carcasses. Also, we note more occurrences of coyotes scavenging on intact coyote carcasses may have been observed had our trials run longer.

Resource availability and the nutritional benefit of carrion may largely determine a species’ willingness to scavenge a particular carcass type, including conspecifics^14,23,54^. For example, Oliva-Vidal et al.^23^ documented extensive red fox cannibalization on fresh and decomposed carcasses, including bone remains, potentially due to food shortages, whereas other studies only observed minimal if any red fox cannibalism^12,28^. Altered carcass trials were deployed in the spring whereas intact trials were conducted in the winter potentially resulting in different resource availability during these trial periods. Differing resource availability as well as the quicker decomposition and depletion of altered carcasses in warmer conditions in the spring may have contributed to more incidents of coyotes cannibalizing on altered carcasses. Future research should investigate the relationship between resource availability and scavenging patterns, including the frequency of cannibalism, by incorporating and assessing precipitation, mast production, and prey abundance data from study areas. It is also possible the fat content on carcasses could influence individuals’ and species’ decisions to scavenge a particular carcass, as bearded vultures have been shown to select more fatty bones when foraging^55^. The subcutaneous fat deposits visible after removing the skin of altered carcasses appeared larger for wild pigs than similarly sized coyotes (Fig. S2), which could lead to scavengers preferring wild pig carrion; however, we did not assess this metric in our study.

Aside from the difference in cannibalism rates between intact and altered carcasses, there was no other indication modifying carcasses resulted in scavengers more readily consuming coyote carrion in comparison to intact trials. This is showcased with raccoons preferring wild pig over coyote carcasses at both intact and altered trials. For instance, raccoons scavenged on both intact paired and unpaired wild pig carcasses, but only scavenged at intact unpaired coyote carcasses. Similarly, at altered trials raccoons only scavenged wild pig carcass types despite visiting an equivalent number of coyote and pig trials. These results generally support our hypothesis that recognition of carcass types is most likely heavily dependent on chemical and not visual cues. This was further exemplified by coyote carcasses persisting significantly longer than wild pig carcasses for both intact and altered trials, demonstrating the removal of external characteristics and associated chemical stimuli did not result in similar scavenging rates between higher and lower trophic level carcass types. However, we had a smaller sample size of altered carcass trials and there was as substantial reduction in scavenger diversity, likely due to rapid depletion times, at altered carcasses compared to intact carcasses. This makes decerning and comparing general patterns challenging and therefore further research should investigate species’ use of sensory recognition mechanisms in the decision process to scavenge.

We distinctly tested assumptions regarding carrion avoidance behaviors and scavenger sensory recognition mechanisms in our study using a robust sample size of a common North American carnivore species. Our findings further support carcass types from different trophic levels are used differently by vertebrate scavengers, and that mammalian carnivore carrion is avoided broadly by other mammal carnivores^12,18,28^. The longer persistence times of carnivore carrion likely plays a role in the transfer of higher trophic level carrion nutrients into soil microbial, plant, and invertebrate communities^17,35,56^. Further, the removal of skin and extremities resulted in altered coyote carcasses persisting for considerably less time than their intact counterpart carcasses, which suggests carcass condition plays an important role in decomposition and carcass depletion processes for carcasses not as readily consumed by vertebrate scavengers. Although warmer temperatures also likely contributed to the shorter persistence of altered coyote carcasses (intact: $\overline{x}=$ 12.03 °C; altered: $\overline{x}=$ 19.48 °C). Altering the visual appearance of carcasses, which made them more similar in appearance, did not result in equal consumption of wild pig and coyote carcasses, as carnivore carrion consistently persisted longer than wild pig carrion. Thus, scavengers are likely capable of responding predominantly to olfactory cues while determining carcass types and associated risks when assessing carrion resources, although visual cues may also play a role. However, future research should investigate the mechanisms and the degree through which species and individuals identify carcass types and how this relates to carrion avoidance behaviors, as these factors have important implications for ecological relationships, nutrient cycling, and zoonotic risks.

## Methods

### Study area

We conducted this research at the Savannah River Site (SRS), a U.S. Department of Energy property in South Carolina, USA. The SRS encompasses 780 km^2^ comprised of 57% pine forest, 21% bottomland hardwood forest, 6% mixed forest, and 16% other (e.g., buildings, roads, open water, etc.). Since its establishment in the 1950s, the SRS has been managed for timber production primarily through planting and maintaining slash pine (*Pinus elliottii*), loblolly pine (*Pinus taeda*), and longleaf pine (*Pinus palustris*) stands. Dominant hardwood species include oak (*Quercus* spp.), hickory (*Carya* spp.), American beech (*Fagus grandifolia*), and American sweetgum (*Liquidambar styraciflua*). There are aquatic reservoirs, several hundred ephemeral wetlands, and swamps associated with bottomlands in the SRS, in addition to five tributaries that drain into the Savannah River, which constitutes the western border of the site. A diversity of scavengers are present including turkey vulture, black vulture (*Coragyps atratus*), Virginia opossum, raccoon, coyote, and wild pig, amongst others^10^.

### Field methods

Using Geographic Information System National Land Cover Database layers in ArcMap 10.7.1 (Redlands, California, USA), we delineated bottomland hardwoods on the SRS that were within 200 m of a road. In the delineated areas we randomly selected 90 locations that were a minimum of 1 km apart, to promote independence among trials, and confirmed site suitability in the field (Fig. S3). Each trial site was used once during the study. An additional 12 locations were randomly selected that met our site suitability requirements to facilitate the inclusion of carcasses received later in the study. These additional locations were a minimum 1 km from trials concluded one month prior, and at least 150 m from carrion trials concluded two months prior (Fig. S3). We established sites within bottomland hardwood forests, given water resources are more readily available in these areas which support a diversity of avian and mammalian scavengers. In addition, we located sites within 200 m of a road to facilitate deployment of carcasses and maintain adequate distance between sites.

We assessed four carcass configurations in our study: coyote alone, wild pig alone, coyote paired with wild pig, and wild pig paired with coyote. Carcass configurations with paired carcasses consisted of deploying both a coyote and wild pig carcass at one location, 6 m apart. A coyote paired with a wild pig (or alternatively a wild pig paired with a coyote) was classified separately from the carcass it was paired with to facilitate the distinction between scavenging events at the two carcasses. We included paired carcass configurations in our study to investigate fine-scale scavenger feeding preferences and assess potential influences carcasses of different trophic levels might have on scavenging dynamics when positioned in close proximity. We conducted scavenging trials for each carcass configuration using intact or altered coyote and wild pig carcasses. Intact carcasses were not modified in any way, whereas we used a scalpel and loppers and followed the case skinning method to remove the skin, fur, feet, and heads of carcasses used in altered carcass trials (Fig. S2). A total of 20 trials was conducted for each carcass configuration using intact carcasses (i.e., 20 coyote, 20 wild pig, 20 coyote paired with wild pig, and 20 wild pig paired with coyote trials; total of 40 coyote and 40 wild pig carcasses). For altered trials, we conducted 14 trials of each carcass configuration using modified carcasses (i.e., 14 coyote, 14 wild pig, 14 coyote paired with wild pig, and 14 wild pig paired with coyote trials; total of 28 coyote and 28 wild pig carcasses). We alternated the assignment of carcass configurations between locations to ensure dispersion throughout the study area.

We secured two Reconyx no-glow, infrared remote sensing cameras (models PC900 Hyperfire or HP2X HyperFire 2, RECONYX, Inc., Holmen, Wisconsin, USA) on the same tree facing 180 degrees in opposite directions at every trial. At paired carcass trials, each camera faced a carcass, either a wild pig or coyote. We used an extra camera at trials with only one carcass to maintain consistency in our experimental design across trial types, and to increase the area monitored and thus our chances of detecting species that visited a trial but did not approach the carcass^57^. We secured carcasses using a non-relaxing snare staked to the ground with rebar ~ 3 m from the camera. Remote cameras facing carcasses were programmed to take motion triggered images in bursts of three photos, one second apart, with a 30 s period between bursts, in addition to a time-lapse photo taken every five minutes. We programmed the second camera at single carcasses in the same manner, excluding time lapse images.

We conducted trials during winter and spring of 2021. All intact carcass trials were conducted simultaneously from early February to mid-March and deployed for 29–35 days. Altered carcass trials were conducted during two sessions, the first from late March until mid-April (22 days) which consisted of 10 trials of each carcass configuration. The second session of altered trials ran from mid-May to early June (21-days) and consisted of 4 trials of each carcass configuration. Each carcass was weighed before deployment, with intact coyote carcasses ranging from 5.45 to 15.50 kg ($\overline{x}=$ 10.74 kg, SD = 2.99 kg) and wild pigs 4.40–19.40 kg ($\overline{x}=$ 11.17 kg, SD = 4.04 kg). Altered coyote carcasses ranged from 4.00 to 12.15 kg ($\overline{x}=$ 8.41 kg, SD = 1.78 kg) and wild pigs from 3.35 to 11.55 kg ($\overline{x}=$ 6.67 kg, SD = 2.31 kg). For both intact and altered trials we attempted to match the masses of paired carcasses, therefore only subadult pigs were used in this study. The average difference in mass for paired carcasses was 0.35 kg for intact pairs and 0.45 kg for altered pairs.

All wild pig carcasses were obtained from the U.S. Department of Agriculture, Wildlife Services and U.S. Forest Service through ongoing removal efforts. Coyote carcasses were opportunistically collected as roadkill or obtained from U.S. Department of Agriculture, Wildlife Services management programs. All carcasses were frozen and completely thawed prior to trial initiation. Altered carcasses were partially thawed to skin and remove distinguishing features and then refrozen to facilitate later deployment. None of the carcasses used in our study were euthanized specifically for our research and all trials were approved by and conducted in compliance with the University of Georgia’s Animal Care and Use Committee (Protocol: A2020 02-029) and were applicable with Animal Research: Reporting In Vivo Experiments (ARRIVE) guidelines. All methods were performed in accordance with relevant guidelines and regulations.

### Image analyses

We reviewed all motion-triggered images and identified species photographed visiting trials. We considered visitations as scavenging events when individuals were photographed consuming or manipulating the carcass and instances as visits when an individual was detected but did not consume any part of the carcass. Time-lapse images were reviewed only when a carcass moved between consecutive visitations without documentation of the animal responsible for disturbing the carcass. If no species was documented moving a carcass, we considered the event as an unknown species scavenging event. We recorded the date and time for every event as well as the number and species of individuals present. A visitation was only considered to be a new event if it occurred more than 10 min from a previous photograph of the same species. We processed all image data in CPW Photo Warehouse ^58^.

### Statistical analyses

For every trial, we determined the time to first investigation, time to first scavenging, carcass persistence time, and vertebrate scavenger community composition and richness. We quantified time to first investigation as the duration from trial initiation until a vertebrate scavenger was detected. This could have been either a visit or scavenging event. We considered a species to be a scavenger if it was documented scavenging carrion at the SRS in Turner et al.^10^ or if it was a known predator. Extra cameras at unpaired carcass trials were reviewed and considered for determining the earliest scavenger detected at unpaired carcass trials. At trials with paired carcasses, the earliest detection of a scavenging species at either carcass was considered the first investigation time for both carcasses present. Time until first scavenging event was determined as the time until a species was first documented scavenging on a carcass, and if a carcass was not scavenged the duration of the trial was used. Lastly, we determined carcass persistence as the time until the carcass was entirely consumed or removed from the field of view of the camera by a scavenger. For carcasses that were not entirely depleted at the end of the trial, the trial end time was used as the carcass persistence time and these data were censored in carcass persistence Kaplan survival analyses (intact carcasses: coyote—16, coyote paired—15, pig—2, pig paired—1; altered carcasses: coyote—11, coyote paired—9, pig—2, pig paired—3).

We initially analyzed intact and altered trials separately, because removing the skin of altered carcasses exposed flesh facilitating easier consumption for invertebrates and vertebrates, thus likely affecting scavenging dynamics^29^. To investigate whether carcass configuration influenced first investigation time, first scavenging time, or carcass persistence, we conducted separate Kaplan–Meier analyses for both intact and altered trials. This allowed us to approximate the probability of an event occurring in a respective time frame (i.e., survival curve). This analysis uses a log-ranked (Mantel-cox) test to investigate differences between survival curves among the four carcass configurations. Carcass size is known to influence carcass depletion times^9,10,27^ and carcass mass varied for both wild pig and coyote carcasses used in our study, although this variation was similar across the two species. Thus, we included carcass mass in Cox-proportional hazards models (analogous to multiple regression models) as a continuous, independent variable to assess differences in carcass persistence times among carcass configurations for intact and altered carcasses. We used the R packages *survival*^59^ and *survminer*^60^ to conduct Kaplan–Meier analyses, to test for differences between survival curves, and to implement Cox proportional hazards models.

We determined summary aspects for every trial including the most frequent scavenger (i.e., species with most scavenging events), and the last scavenger to consume the remaining flesh on a carcass or removed it if it was entirely scavenged before the end of the trial. These summary data are observational and were not statistically analyzed.

To explore scavenging species composition across the four carcass configurations we conducted separate correspondence analysis (CA) for intact and altered trials. We used the packages *ca*^61^ and *factoextra*^62^ and used the contribution biplot scaling to display the solutions^63^. In addition, we determined scavenger species richness for each trial and calculated means within carcass configurations to further assess differences in scavenging communities. We considered species richness as the number of species that scavenged on a carcass. We conducted separate Poisson generalized linear models with a log link for intact and altered trials to assess the influence of carcass configuration and carcass mass on scavenging species richness. To evaluate for overdispersion we used a chi-squared goodness-of-fit test for each Poisson regression analysis. We also conducted separate post-hoc Kruskal–Wallis tests, followed by a Dunn’s multiple comparison test using Bonferroni corrected *P*-values, for intact and altered trials to investigate whether combined scavenger species richness of both coyote and wild pig carcasses at a paired trial sites was different from richness of each unpaired carcass configuration. We calculated combined species richness by adding together the species that scavenged the coyote or wild pig carcass at a paired site; species that scavenged on both carcasses were only counted once.

We fit separate GLMs with a Gamma distribution and a log link for pig and coyote trials to determine whether altered carcasses were depleted quicker than intact carcasses. Given intact and altered carcass trials were conducted in different seasons we considered temperature as an independent variable in the models. We obtained temperature data from a nearby weather station in Augusta, GA and calculated the mean temperature (°C) during each carcass trial using daily averages (WBAN: 03820; www.ncdc.noaa.gov). Instead of performing these tests across the four carcass configurations we combined paired and unpaired coyote carcass data as well as the paired and unpaired pig carcass data. We generated diagnostic plots using the R package *DHARMa* to assess model assumptions including deviations from the expected distribution, residuals dispersion, and quantile regression^33^. In addition, we conducted a Fisher’s exact test, due to a small sample size, to investigate whether carcass condition (intact vs. altered) influenced the number of coyote trials cannibalized using the combined coyote (paired and unpaired) carcass trial data. We performed all of our analyses in R Version 4.0.0^64^.

## Supplementary Information

Supplementary Information 1.

Supplementary Information 2.

## Data Availability

All data generated or analyzed during this study are included in this published article (and its Supplementary Information files).
