# Supplementary material for: Carcass appearance does not influence scavenger avoidance of carnivore carrion
Source: Sci Rep. 2022 Nov 7;12:18842. doi: 10.1038/s41598-022-22297-8 (PMC9640519; doi:10.1038/s41598-022-22297-8)
Supplement: Supplementary file 2 — Supplementary Information 2. [file 41598_2022_22297_MOESM2_ESM.docx]

**Article title:**

Carcass appearance does not influence scavenger avoidance of carnivore carrion

**Journal name:**

Scientific Reports

**Author names:**

Miranda J. Butler-Valverde, Travis L. DeVault, Olin E. Rhodes Jr., James C. Beasley

**Affiliation and email address of corresponding author:**

Savannah River Ecology Laboratory, Warnell School of Forestry and Natural Resources, University of Georgia, PO Drawer E, Aiken, SC 29802, USA, mbutlervalverde@gmail.com

Table S1. The percent occurrence each species visited in comparison to percent occurrence scavenged (separated by “-“) for each intact carcass type based on trials conducted in February - June 2021 at the Savannah River Site in Aiken, SC, USA.

|  | Intact Carcass Configuration | | | |  |
| --- | --- | --- | --- | --- | --- |
|  | Coyote | Coyote paired | Wild pig | Wild pig paired | Overall |
| Species | 18 trials | 19 trials | 20 trials | 19 trials | 76 trials |
| **Avian** |  |  |  |  |  |
| Barred owl | 5.6 - 0 | 5.3 - 0 | 0 - 0 | 5.3 - 5.3 | 3.9 - 1.3 |
| *Strix varia* | |  |  |  |  |
| Black vulture | 16.7 - 16.7 | 42.1 - 42.1 | 15.0 - 15.0 | 36.8 - 31.6 | 2.8 - 26.3 |
| *Coragyps atratus* | |  |  |  |  |
| Crow | 5.6 - 5.6 | 5.3 - 5.3 | 5.0 - 0 | 5.3 - 0 | 5.3 - 2.6 |
| *Corvus* spp. | |  |  |  |  |
| Great-horned owl | 16.7 - 11.1 | 10.5 - 0 | 0 - 0 | 10.5 - 0 | 9.2 - 2.6 |
| *Bubo virginianus* | |  |  |  |  |
| Red-tailed hawk | 5.6 - 5.6 | 10.5 - 10.5 | 5.0 - 0 | 10.5 - 5.3 | 7.9 - 5.3 |
| *Buteo jamaicensis* | |  |  |  |  |
| Turkey vulture | 94.4 - 94.4 | 100.0 - 100.0 | 45.0 - 45.0 | 100.0 - 94.7 | 84.2 - 82.9 |
| *Cathartes aura* | |  |  |  |  |
| **Mammal** |  |  |  |  |  |
| Bobcat | 22.2 - 0 | 36.8 - 0 | 35.0 - 15.0 | 42.1 - 5.3 | 34.2 - 5.3 |
| *Lynx rufus* | |  |  |  |  |
| Coyote | 94.4 - 0 | 100.0 - 5.3 | 100.0 - 85.0 | 100.0 - 100.0 | 98.7 - 48.7 |
| *Canis latrans* | |  |  |  |  |
| Raccoon | 77.8 - 11.1 | 84.2 - 0 | 80.0 - 5.0 | 78.9 - 10.5 | 80.3 - 6.6 |
| *Procyon lotor* | |  |  |  |  |
| Southern flying squirrel | 5.6 - 0 | 5.3 - 0 | 10.0 - 5.0 | 10.5 - 5.3 | 7.9 - 2.6 |
| *Glaucomys volans* | |  |  |  |  |
| Virginia opossum | 77.8 - 77.8 | 89.5 - 73.7 | 55.0 - 45.0 | 84.2 - 63.2 | 76.3 - 64.5 |
| *Didelphis virginiana* | |  |  |  |  |
| Wild pig | 94.4 - 0 | 78.9 - 5.3 | 80.0 - 0 | 73.7 - 0 | 81.6 - 1.3 |
| *Sus scrofa* | |  |  |  |  |

Table S2. The percent occurrence each species visited in comparison to percent occurrence scavenged (separated by “-“) for each altered carcass configuration based on trials conducted in February - June 2021 at the Savannah River Site in Aiken, SC, USA.

|  | Altered Carcass Configuration | | | |  |
| --- | --- | --- | --- | --- | --- |
|  | Coyote | Coyote paired | Wild pig | Wild pig paired | Overall |
| Species | 14 trials | 14 trials | 14 trials | 14 trials | 56 trials |
| **Avian** |  |  |  |  |  |
| Black vulture | 92.9 - 85.7 | 64.3 - 64.3 | 28.6 - 28.6 | 64.3 - 57.1 | 62.5 - 58.9 |
| *Coragyps atratus* | |  |  |  |  |
| Turkey vulture | 100.0 - 100.0 | 100.0 - 100.0 | 92.9 - 92.9 | 100.0 - 100.0 | 98.2 - 98.2 |
| *Cathartes aura* | |  |  |  |  |
| **Mammal** |  |  |  |  |  |
| Coyote | 92.9 - 35.7 | 92.9 - 7.1 | 100.0 - 71.4 | 92.9 - 50.0 | 94.6 - 41.1 |
| *Canis latrans* | |  |  |  |  |
| Raccoon | 64.3 - 0 | 57.1 - 0 | 57.1 - 7.1 | 57.1 - 14.3 | 58.9 - 5.4 |
| *Procyon lotor* | |  |  |  |  |
| Virginia opossum | 85.7 - 85.7 | 78.6 - 78.6 | 21.4 - 21.4 | 78.6 - 64.3 | 66.1 - 62.5 |
| *Didelphis virginiana* | |  |  |  |  |
| Wild pig | 85.7 - 0 | 57.1 - 7.1 | 50.0 - 0 | 57.1 - 0 | 62.5 - 1.8 |
| *Sus scrofa* | |  |  |  |  |
| **Reptile** |  |  |  |  |  |
| American alligator | 7.1 - 7.1 | 7.1 - 7.1 | 0 - 0 | 7.1 - 7.1 | 5.4 - 5.4 |
| *Alligator mississippiensis* | | |  |  |  |

Table S3. The number of events and percent occurrence (in paratheses) each species was observed scavenging each intact carcass configuration based on trials conducted in February - June 2021 at the Savannah River Site in Aiken, SC, USA.

|  | Intact Carcass Configuration | | | |  | |
| --- | --- | --- | --- | --- | --- | --- |
|  | Coyote | Coyote paired | Wild pig | Wild pig paired | | Overall |
| Species | 668 events | 613 events | 458 events | 567 events | | 2,306 events |
| **Avian** |  |  |  |  | |  |
| Barred owl | 0 (0) | 0 (0) | 0 (0) | 2 (0.4) | | 2 (0.1) |
| *Strix varia* |  |  |  |  | |  |
| Black vulture | 3 (0.4) | 38 (6.2) | 11 (2.4) | 29 (5.1) | | 81 (3.5) |
| *Coragyps atratus* |  |  |  |  | |  |
| Crow | 1 (0.1) | 1 (0.2) | 0 (0) | 0 (0) | | 2 (0.1) |
| *Corvus* spp |  |  |  |  | |  |
| Great-horned owl | 5 (0.7) | 0 (0) | 0 (0) | 0 (0) | | 5 (0.2) |
| *Bubo virginianus* |  |  |  |  | |  |
| Red-tailed hawk | 1 (0.1) | 3 (0.5) | 0 (0) | 14 (2.5) | | 18 (0.8) |
| *Buteo jamaicensis* |  |  |  |  | |  |
| Turkey vulture | 147 (22.0) | 293 (47.8) | 62 (13.5) | 174 (30.7) | | 675 (29.3) |
| *Cathartes aura* |  |  |  |  | |  |
| **Mammal** |  |  |  |  | |  |
| Bobcat | 0 (0) | 0 (0) | 56 (12.2) | 11 (1.9) | | 67 (2.9) |
| *Lynx rufus* |  |  |  |  | |  |
| Coyote | 0 (0) | 1 (0.2) | 122 (26.6) | 112 (19.8) | | 235 (10.2) |
| *Canis latrans* |  |  |  |  | |  |
| Raccoon | 14 (2.1) | 0 (0) | 4 (0.9) | 9 (1.6) | | 27 (1.2) |
| *Procyon lotor* |  |  |  |  | |  |
| Southern flying squirrel | 0 (0) | 0 (0) | 1 (0.2) | 6 (1.1) | | 7 (0.3) |
| *Glaucomys volans* |  |  |  |  | |  |
| Virginia opossum | 490 (73.4) | 276 (45.0) | 200 (43.7) | 203 (35.8) | | 1,169 (50.7) |
| *Didelphis virginiana* |  |  |  |  | |  |
| Wild pig | 0 (0) | 1 (0.2) | 0 (0) | 0 (0) | | 1 (0.0) |
| *Sus scrofa* |  |  |  |  | |  |
| **Unknown** | 8 (1.2) | 1 (0.2) | 2 (0.4) | 7 (1.2) | | 17 (0.7) |

Table S4. The number of events and percent occurrence (in parentheses) each species was observed scavenging each altered carcass configuration based on trials conducted in February - June 2021 at the Savannah River Site in Aiken, SC, USA.

|  | Altered Carcass Configuration | | | |  | |
| --- | --- | --- | --- | --- | --- | --- |
|  | Coyote | Coyote paired | Wild pig | Wild pig paired | | Overall |
| Species | 339 events | 465 events | 142 events | 371 events | | 1,317 events |
| **Avian** |  |  |  |  | |  |
| Black vulture | 26 (7.7) | 19 (4.1) | 5 (3.5) | 17 (4.6) | | 67 (5.1) |
| *Coragyps atratus* |  |  |  |  | |  |
| Turkey vulture | 157 (46.3) | 236 (50.8) | 74 (52.1) | 182 (49.1) | | 649 (49.3) |
| *Cathartes aura* |  |  |  |  | |  |
| **Mammal** |  |  |  |  | |  |
| Coyote | 8 (2.4) | 1 (0.2) | 27 (19.0) | 16 (4.3) | | 52 (3.9) |
| *Canis latrans* |  |  |  |  | |  |
| Raccoon | 0 (0) | 0 (0) | 4 (2.8) | 9 (2.4) | | 13 (1.0) |
| *Procyon lotor* |  |  |  |  | |  |
| Virginia opossum | 144 (42.5) | 203 (43.7) | 30 (21.1) | 142 (38.3) | | 519 (39.4) |
| *Didelphis virginiana* |  |  |  |  | |  |
| Wild pig | 0 (0) | 1 (0.2) | 0 (0) | 0 (0) | | 1 (0.1) |
| *Sus scrofa* |  |  |  |  | |  |
| **Reptile** |  |  |  |  | |  |
| American alligator | 1 (0.3) | 5 (1.1) | 0 (0) | 3 (0.8) | | 9 (0.7) |
| *Alligator mississippiensis* |  |  |  |  | |  |
| **Unknown** | 3 (0.9) | 0 (0) | 2 (1.4) | 2 (0.5) | | 7 (0.5) |

Table S5. Generalized linear model (specifying Poisson and log link) results for scavenger species richness at (a) intact carcasses and (b) altered carcasses trials conducted in February - June 2021 at the Savannah River Site in Aiken, SC, USA.

| a) Intact Carcasses | | | | | | |
| --- | --- | --- | --- | --- | --- | --- |
|  |  |  | 95% Confidence Limits | |  |  |
| Predictor | Estimate | *SE* | Lower | Upper | Z value | *P* value |
| Intercept | 1.059 | 0.255 | 0.549 | 1.552 | 4.144 | 0.000 |
| Coyote | -0.373 | 0.204 | -0.779 | 0.022 | -1.831 | 0.067 |
| Coyote paired | -0.282 | 0.195 | -0.670 | 0.098 | -1.445 | 0.148 |
| Wild pig | -0.421 | 0.204 | -0.827 | -0.026 | -2.067 | 0.039 |
| Weight | 0.010 | 0.021 | -0.031 | 0.052 | 0.489 | 0.625 |
|  |  |  |  |  |  |  |
| b) Altered Carcasses | | | | | | |
|  |  |  | 95% Confidence Limits | |  |  |
| Predictor | Estimate | *SE* | Lower | Upper | Z value | *P* value |
| Intercept | 0.953 | 0.362 | 0.230 | 1.650 | 2.632 | 0.008 |
| Coyote | 0.042 | 0.230 | -0.409 | 0.496 | 0.181 | 0.857 |
| Coyote Paired | -0.109 | 0.227 | -0.558 | 0.337 | -0.479 | 0.632 |
| Wild pig | -0.258 | 0.245 | -0.745 | 0.220 | -1.052 | 0.293 |
| Weight | 0.017 | 0.044 | -0.071 | 0.103 | 0.375 | 0.708 |

Table S6. GLM results assessing carcass condition (i.e., intact and altered) and mean trial temperature effects on carcass persistence times for trials conducted in February - June 2021 at the Savannah River Site in Aiken, SC, USA.

| Model | Equation | Variable | Estimate | Std. Error | *t* value | *P* value |
| --- | --- | --- | --- | --- | --- | --- |
| Coyote | glm(formula = CarcassPersistence ~ CarcassCondition + MeanTemp, family = Gamma(link="log"), data=JoinedCoyote) | Intercept | 7.06 | 0.37 | 19.05 | <0.0001 |
|  |  | Carcass Condition - Intact | 0.27 | 0.08 | 3.35 | 0.001 |
|  |  | Mean temp | -0.01 | 0.01 | -2.30 | 0.02 |
| Wild pig | glm(formula = CarcassPersistence ~ CarcassCondition + MeanTemp, family = Gamma(link="log"), data=JoinedPig) | Intercept | 4.64 | 1.67 | 2.79 | 0.007 |
|  |  | Carcass Condition - Intact | 0.63 | 0.35 | 1.78 | 0.08 |
|  |  | Mean temp | 0.02 | 0.02 | 0.64 | 0.52 |

**
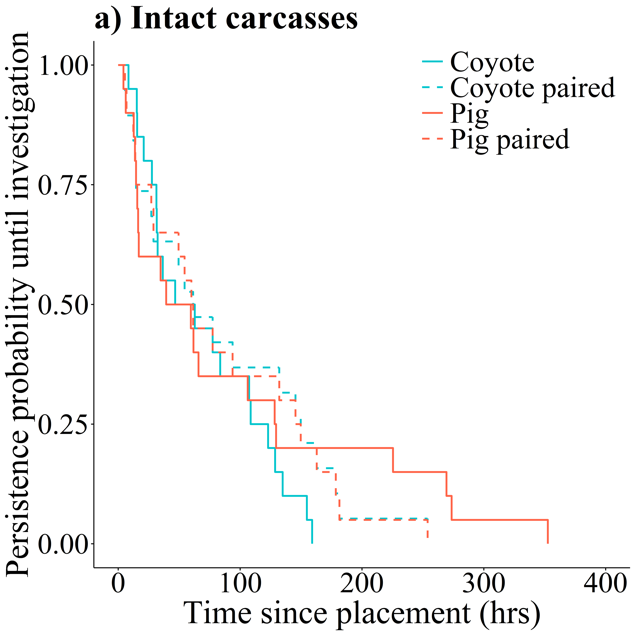

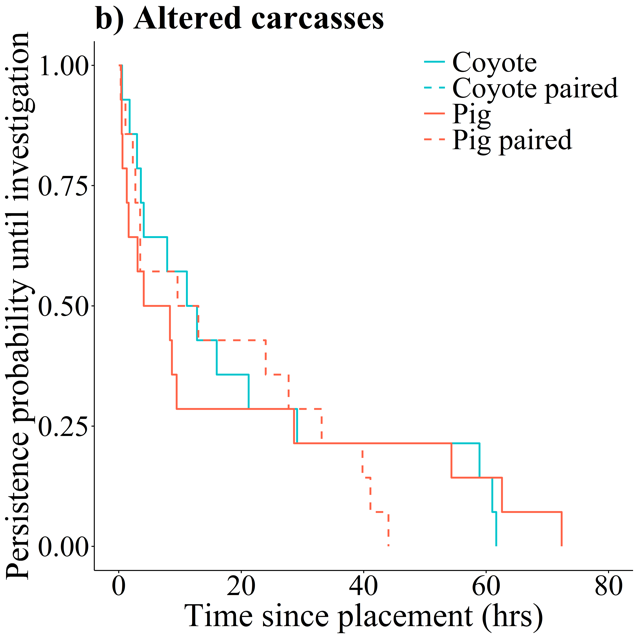
**

**
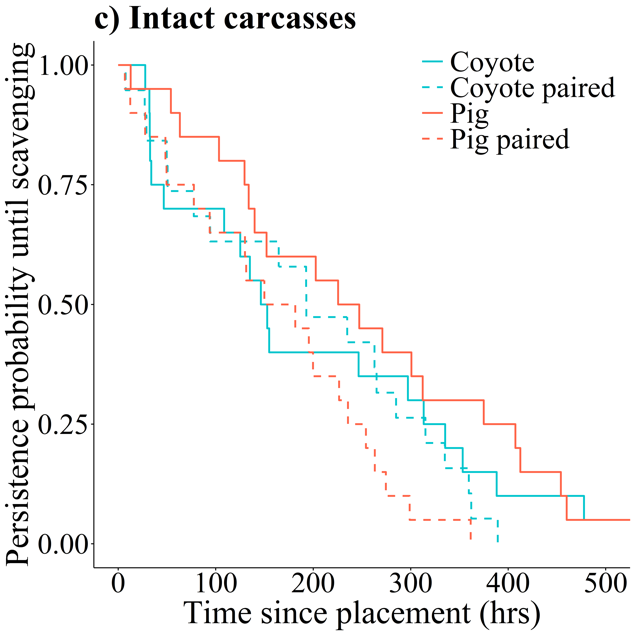

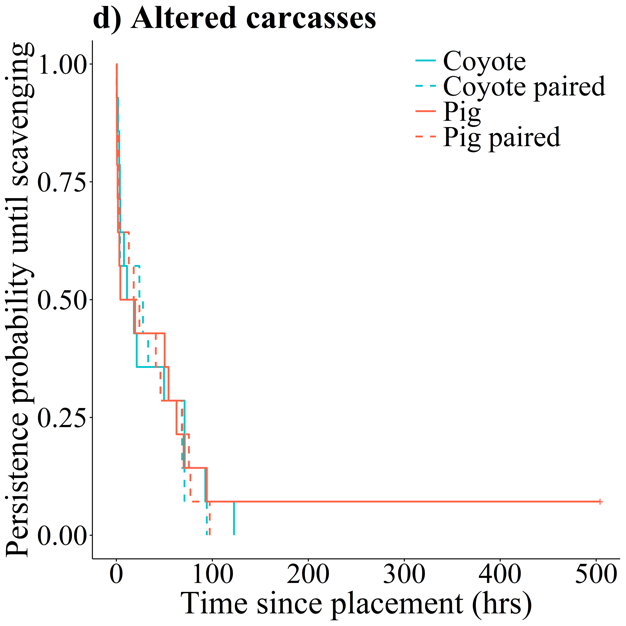
**

Figure S1. Kaplan-Meir survival function estimate of the probability of the four carcass configurations persisting on the landscape until first investigated for (a) intact carcass trials and (b) altered carcass trials and until first scavenged for (c) intact carcass trials and (d) altered carcass trials. Carcasses were deployed February - June 2021 at the Savannah River Site, Aiken, SC, USA. Censoring is indicated by the plus sign tick mark.

**
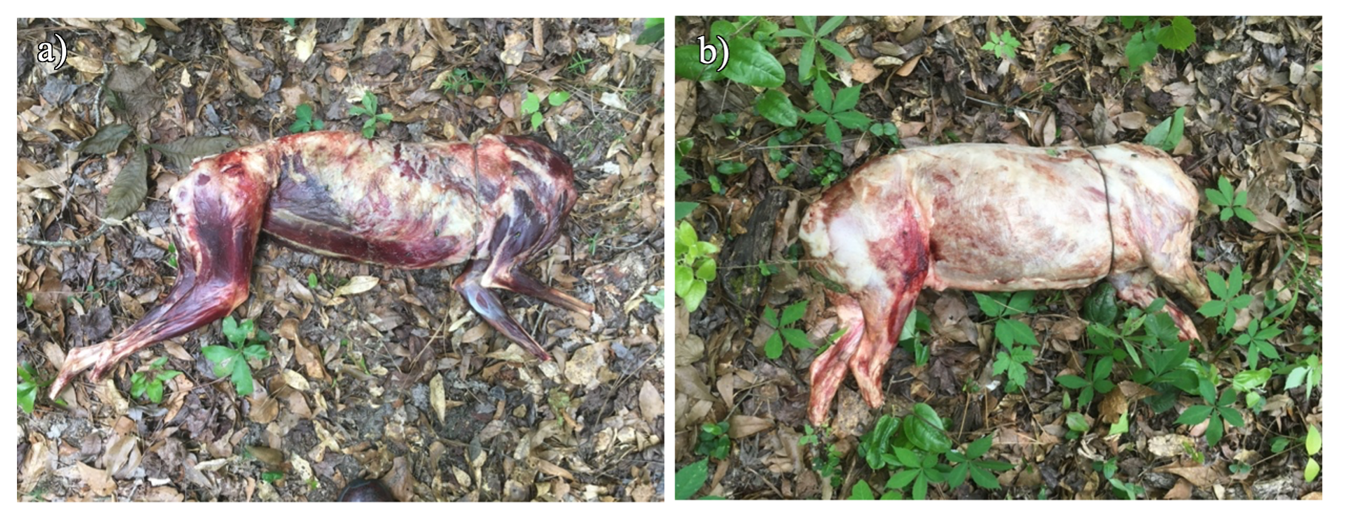
**

Figure S2. Altered carcasses used in scavenging research at the Savannah River Site, Aiken, SC, USA. Pictures show an altered coyote carcass a), and an altered pig carcass b).


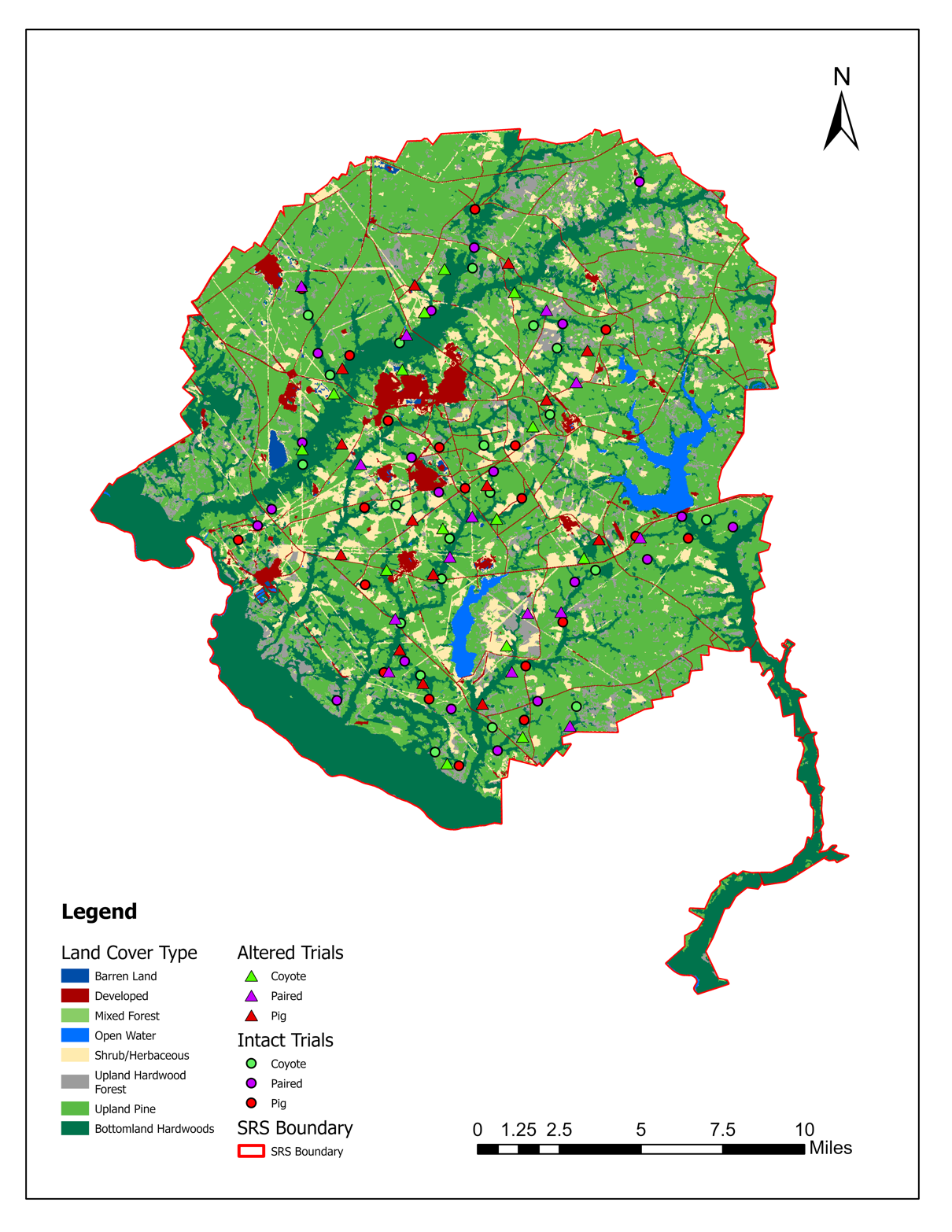


Figure S3. Overall study area with locations altered and intact carcass trials were conducted from February - June 2021 at the Savannah River Site, Aiken, SC, USA.
